# Symptom benchmarks of improved quality of life among military veterans with PTSD^☆^

**DOI:** 10.1016/j.xjmad.2026.100183

**Published:** 2026-05-28

**Authors:** Paula P. Schnurr, Carole A. Lunney, Kathleen M. Chard, Josef I. Ruzek, Brian P. Marx, Thomas M. Crow

**Affiliations:** a National Center for PTSD (Executive Division), White River Junction, VT and Department of Psychiatry, Geisel School of Medicine at Dartmouth, Hanover, NH, United States; b Department of Psychology, University of Calgary, Calgary, Canada; c Cincinnati VA Medical Center and University of Cincinnati, United States; d Department of Psychiatry and Behavioral Sciences, Stanford University, and Palo Alto University and Lyda Hill Institute of Human Resilience, University of Colorado, Colorado Springs, United States; e National Center for PTSD (Behavioral Science Division), VA Boston Healthcare System, and Boston University Chobanian and Avedisian School of Medicine, United States; f National Center for PTSD (Behavioral Science Division), VA Boston Healthcare System, and Boston University Chobanian and Avedisian School of Medicine, United States

**Keywords:** PTSD, Trauma-focused therapy, Veterans, Clinical significance, Quality of life

## Abstract

**Objective:**

To optimize treatment outcome and promote recovery, it is necessary to understand how clinical indicators of improvement translate into meaningful differences in the lives of individuals with posttraumatic stress disorder (PTSD). Prior research has suggested that treatment response alone is insufficient and that more substantial improvement is needed, but data are limited. This study addressed the gap through secondary analysis of a pragmatic comparative effectiveness trial of Cognitive Processing Therapy (CPT) and Prolonged Exposure (PE) in veterans.

**Methods:**

Participants were 597 male and female US military veterans who were randomized to receive CPT or PE. PTSD symptom change from pre- to posttreatment was categorized into four mutually exclusive categories: No Response, Response, Loss of Diagnosis, and Remission. Analyses compared each category to the prior one to identify the effect of attaining a higher benchmark on experiencing clinically meaningful improvements and good endpoints (no/little impairment in functioning or ≥70% on a QoL scale) on clinician-rated and self-reported measures.

**Results:**

Response was associated with improvement on most outcomes, but with a good endpoint on only one. Loss of Diagnosis added little benefit beyond response. Remission was associated with increased likelihood of meaningful improvement (OR=2.31–23.65, lowest *p* < .001) and a good endpoint (OR=3.37–13.50, lowest *p* < .001) on all measures.

**Conclusions:**

Response without more substantial improvement following PTSD treatment is insufficient for helping patients achieve good QoL. Findings support the position that focusing on more than symptom change and response alone is necessary when setting treatment goals for individual patients and for evaluating treatments.

## Highlights

• This study examined how PTSD symptom change following treatment relates to quality of life (QoL).
• Remission, but not response or loss of diagnosis, was associated with a good QoL endpoints.
• Focusing on more than symptom change is necessary to help patients attain good QoL.

## 1 Introduction

There are a range of effective treatments for posttraumatic stress disorder (PTSD), including trauma-focused psychotherapies such as Cognitive Processing Therapy (CPT), Prolonged Exposure (PE), Eye Movement Desensitization and Reprocessing, and Written Exposure Therapy, as well as select medications [1], [2]. Most people respond. For example, in a recent comparative effectiveness trial of CPT and PE in a sample of US veterans, there were large effect sizes observed for symptom decrease from before to after treatment, and 60% of CPT participants and 73% of PE participants responded [3].

Reduction in symptom severity is typically the primary outcome in treatment trials for PTSD and other mental disorders, but symptom change does not indicate how many people have a meaningful response. In the study comparing CPT with PE, despite the large pre-post improvements, 28%−40% of participants no longer met diagnostic criteria and 13%−20% remitted, in CPT and PE, respectively [3]. To optimize treatment outcome, it is necessary to understand how indicators of symptom improvement are associated with meaningful improvements. Measures such as clinically significant change [4], [5], which are based on statistical significance and similarity to normative samples, are often used to capture clinical significance and are helpful indicators of how individuals (versus groups) change. However, symptom change does not necessarily reflect change in QoL, which may not improve meaningfully despite substantial symptom change or may improve meaningfully despite little change. In 2001, Kazdin raised this and other concerns about the measurement of clinical significance and offered recommendations for research to address those concerns [6].

One recommendation was a novel approach to defining clinical significance by validating measures of clinical significance using indicators of QoL. In PTSD, several prior studies did this by comparing categorical measures of symptom change with change in QoL. A randomized clinical trial comparing PE and Present-Centered Therapy for female US veterans and soldiers used 4 mutually exclusive categories: no treatment response, treatment response only, loss of diagnosis (treatment response plus no longer meeting diagnostic symptom and severity criteria), and remission (loss of diagnosis plus a score lower than an established severity threshold) [7]. The comparisons between successive groups allowed for testing the value added of each successive clinical benchmark. Response was associated with meaningful change in functioning and overall QoL, but loss of diagnosis (response plus no longer meeting diagnostic criteria) was associated with higher likelihood of meaningful change and achieving a good endpoint in QoL; remission conferred only limited further improvement. Similar findings were reported in a study of Australian veterans who received outpatient treatment for PTSD [8]. A study of civilians who received CPT found that both no longer meeting diagnostic criteria and remission were associated with improved overall QoL and functioning, but used inclusive groups for comparison e.g., no response vs. response/loss of diagnosis/remission, no response/response vs. loss of diagnosis/remission [9]. Lastly, a study of US veterans who received CPT or WET compared nonresponders with all responders and found that response was associated with improved functioning [10].

Data from the two studies that directly compared response categories [6], [7] suggest that remission is not necessary and that loss of diagnosis (rigorously defined) is sufficient to help patients attain good QoL, but replicating these findings in larger, more heterogeneous samples using DSM−5 diagnostic criteria [11] is important given the limited data. Therefore, we performed secondary analyses of data from a large pragmatic comparative effectiveness trial of CPT and PE in US veterans [3] that found statistically but not clinically significant differences favoring PE for decreasing symptom severity; however, PE had statistically and clinically larger effects than CPT on response, loss of diagnosis, and remission. In the present study, we compared mutually exclusive groups to pinpoint the specific benefit of each category of symptom improvement. Based on the findings of prior studies that used this approach [6], [7], we expected that loss of diagnosis would be associated with optimal response, defined as clinically meaningful improvement and attaining a good endpoint (no/little impairment in functioning or ≥70% on a QoL scale).

## 2 Method

The study was a parallel two-arm trial in which participants at 17 VA Medical Centers were randomized to receive PE or CPT [12]. The study was approved by VA’s Central Institutional Review Board. Participants gave written informed consent before participation.

### 2.1 Participants

Participants were 597 male and female veterans with current military-related PTSD according to DSM−5 [11] and symptom severity of ≥ 25 on the Clinician-Administered PTSD Scale−5 (CAPS−5) [13]. They were selected from 916 participants in the primary study because they had pre- and post-treatment data. Participants agreed to not receive other PTSD psychotherapy during study treatment. Medications for PTSD and other mental or physical conditions, psychotherapy for other problems, brief visits with an existing therapist, and participation in self-help groups were allowed. Individuals taking medication were initially required to have no changes in drugs or dosage for 2 months before entry; during the trial, the duration was reduced to 1 month to increase recruitment. Exclusion criteria were substance dependence not in remission for 1 month; current psychotic symptoms or mania; current suicidal or homicidal intent that required immediate attention; or moderate to severe cognitive impairment.

### 2.2 Measures

Measures to assess demographic characteristics and eligibility were administered at baseline. We assessed PTSD using the CAPS−5 [13], a clinician-administered structured interview that has excellent psychometric properties. The 20 symptoms of PTSD [10] (are rated on a 0–4 scale and are summed for total severity; symptoms are counted as present if ≥2. For diagnosis, symptoms had to occur at least monthly with moderate intensity (the “1/2” rule) and overall severity had to be ≥ 25 [13]. Other Axis 1 diagnoses were measured using the Structured Clinical Interview for DSM−5 (SCID) [14]. Independent doctoral-level clinicians at two centralized sites who were masked to treatment condition conducted CAPS−5 and SCID−5 telephone interviews. Inter-rater reliability was high for both interview measures. In the present sample, internal consistency for the CAPS−5 was high (Cronbach’s α =.90). As in the main study [3], we calculated categorical response indicators for the CAPS−5 from baseline to posttreatment as: Response (≥10 point improvement in severity), Loss of Diagnosis (response, no longer meeting DSM−5 symptom criteria, and severity <25), and Remission (Loss of Diagnosis and severity <12). This approach to categorization had previously been validated for the prior version of the CAPS using measures of QoL [7].

We assessed QoL using several measures. Clinician-rated social and occupational functioning were assessed using the single items for these constructs on the CAPS−5, which are scored on a 0–4 scale; higher scores indicate poorer functioning. Overall self-reported functional impairment was assessed using a modified 8-item version of the 12-item World Health Organization Disability Adjustment Scale-II (WHODAS-II) [15], with items for mobility/self-care (standing, walking, washing, and getting dressed) removed to enhance sensitivity to change in items more relevant to a mental health intervention. Items were rated on a 1–5 scale and averaged to create an overall measure of impairment; higher scores indicate greater impairment. Domains of QoL were assessed using the four subscales (physical, psychological, social, and environmental) of the 12-item World Health Organization Quality of Life-BREF (WHOQOL-Bref) [16], with items rated on a 5-point scale, and summed within subscales and transformed using the recommended 0–100 scoring representing the percentage of maximum possible score for each subscale. Higher scores indicate better QoL. Cronbach’s αs for all multi-item QoL measures were as follows: WHODAS (.88), WHOQOL-Bref physical (.85), WHOQOL-Bref psychological (.85), WHOQOL-Bref social (.72), and WHOQOL-Bref environmental (.83).

There were limited data to inform definitions of clinically meaningful improvement and a good endpoint on the QoL measures. Following the categorization used in a prior study [6], clinically meaningful improvement on each CAPS−5 item was defined as a 1-point categorical change toward better functioning and a good endpoint was defined as no (0) or minimal (1) impairment. A similar approach was used to define clinically meaningful improvement and a good endpoint on the WHODAS-II using the item average so that clinically meaningful improvement was defined as a decrease of 1 or more points and a good endpoint was defined as an item average of ≤ 2 (no or minimal impairment). The 0–100 scoring on the WHOQOL-Bref required a different approach. We translated the definition of clinically meaningful improvement used for the CAPS−5 (10 points, or 12.5% of the scale range of 0–80) to 12.5 points on the WHOQOL-Bref and defined a good endpoint as 70 or higher. There were no relevant normative scores or validated definitions of a good endpoint on the WHOQOL-Bref, but a general population study of Australian adults found scores of 69.6–75.1 among participants who rated their health as good [17].

### 2.3 Treatment

Treatment was delivered in outpatient clinics according to a flexible dosing protocol. A standard dose was 12 weekly sessions, but participants could finish in 10 or 11 sessions if they showed an early response or could receive up to two additional sessions if they had an inadequate response after 12 sessions. The original study found that participants who received PE were more likely to finish early and participants who received CPT were more likely to receive 12 sessions. Receipt of extra sessions was rare (7.6% in CPT, 6.8% in PE). Because this was a pragmatic trial, CPT was delivered in standard 60-minute sessions and PE was delivered in standard 90-minute sessions. CPT and PE did not differ in global ratings of adherence or competence [3].

CPT consists of cognitive therapy and writing two trauma accounts, which is now an optional component in the newest version of CPT [18]. Participants wrote an account of their index stressor that they read to themselves and to therapists after sessions 3 and 4. Most sessions help patients challenge their beliefs through Socratic Dialogue and daily worksheets. The initial focus is on examining beliefs caused by hindsight bias, just-world violations, and self-blame or erroneous other-blame, which then shifts to overgeneralized beliefs about self, others and the world. Patients write statements about the causes and impact of their trauma before and after treatment so they can see changes in their thinking.

PE consists of in-vivo exposure, imaginal exposure, and processing the imaginal exposure [19]. In-vivo exposure involves gradually and systematically approaching distressing trauma-related situations, places, and people that have been avoided, remaining in the situation until distress reduces by half. Imaginal exposure involves repeated revisiting of the memory and recounting aloud the traumatic event(s) in detail, while vividly imagining the event(s). Treatment sessions are audio-recorded and patients are asked to listen to recordings daily. Psychoeducation and controlled breathing exercises are also included.

### 2.4 Statistical Analysis

R 4.5.2 [20] was used for all analyses. For descriptive analyses comparing the PTSD response groups, we used one-way analysis of variance (ANOVA) for continuous variables and Pearson's χ^2^ tests for dichotomous variables. We applied the Tukey-Kramer adjustment for continuous variables and the Holm adjustment for dichotomous variables in the post-hoc pairwise comparisons of demographic and clinical characteristics between all possible pairs of the four PTSD symptom change benchmark categories. For variables with a statistically significant omnibus test, we report the results of these pairwise comparisons, where groups not sharing a common subscript differ from one another (*p* > .05).

We conducted separate logistic regressions predicting good endpoint and meaningful change in each QoL outcome from PTSD symptom change group at posttreatment, controlling for initial PTSD severity and the initial score on that outcome. We used sequential coding [21] for PTSD symptom change benchmark categories, so that each resulting odds ratio (OR) represents the comparison between successive categories (Response vs. No Response, Loss of Diagnosis vs. Response, and Remission vs. Loss of Diagnosis). We examined whether treatment type moderated outcomes, but data are presented collapsed across treatments because only 1 of the 42 possible interactions was statistically significant. In the PE group, response compared with non-response was associated with a higher probability of meaningful change on the WHOQOL-Bref physical health subscale (OR = 5.29, 95%CI [1.18, 26.07], *p* = .033).

## 3 Results

Most participants showed clinically meaningful improvement in PTSD symptoms: 11% responded, 28% lost their PTSD diagnosis, and 23% remitted; 38% did not respond (Table 1). The average amount of decrease on the CAPS−5 ranged from 1.4 points in nonresponders to 30.1 points in remitters.

**Table 1:** Clinical Change from Pre- to Posttreatment.

Clinical change	*n*	%	*M*	*SD*	Range
No Response	225	37.7	−1.4	6.13	+19 to −9
Response	68	11.4	−15.0	4.62	−10 to −27
Loss of Diagnosis	168	28.1	−20.9	7.39	−10 to −45
Remission	136	22.8	−30.1	7.78	−14 to −57

*Note*. Clinical change was determined using the Clinician-Administered PTSD Scale (CAPS). Response was defined as a reduction of 10 or more points. Loss of Diagnosis was defined as Response plus no longer meeting CAPS “1/2” symptom criteria and having a severity score < 25. Remission was defined as Loss of Diagnosis plus a severity score < 12.

Table 2 displays baseline demographic and clinical characteristics. On average, participants were in their mid-40s (range = 23–79 years). Most were male, had at least some college and were married/cohabitating. The majority (70%) were White, 23% were Black, and 11% were other or multiple races; 14% were Hispanic. There were few demographic differences among groups. The No Response group was older than the Response and Loss of Diagnosis groups. The No Response and Response groups were more likely than the Remission group to be Black. The Remission group was more likely than all other groups to be White, as was the Loss of Diagnosis group relative to the No Response group. Clinical characteristics were more robust predictors, although some response patterns were nonlinear, e.g., past only comorbid psychiatric disorder was higher in the Remission group versus the Loss of Diagnosis group, and PTSD severity was highest in the Remission group relative to all other groups. For all other clinical characteristics, the remission group had better functioning and overall QoL than all other groups, which did not differ from one another.

**Table 2:** Pretreatment Demographic and Clinical Characteristics of Response Subgroups.

Variable	Total	No Response	Response	Loss of Diagnosis	Remission	Omnibus Test^1^
	N = 597	N = 225	N = 68	N = 168	N = 136	
Female gender (%)	20.9	17.3	17.8	22.0	25.7	χ^2^(3)= 4.23, *p* = .0238
	(474)	(39)	(12)	(37)	(136)	
Age (*M*)	46.9	49.7_b_	43.4_a_	44.3_a_	47.1_ab_	*F*(3, 593)= 6.98, *p* < .001
	(13.6)	(13.5)	(11.6)	(13.2)	(14.5)	
Post-high-school education (%)	84.1	83.1	92.6	84.5	80.9	χ^2^(3)= 4.95, *p* = .175
	(502)	(187)	(63)	(142)	(110)	
Race: white (%)	70.2	59.6_b_	63.2_ab_	73.2_a_	87.5_c_	χ^2^(3)= 33.94, *p* < .001
	(419)	(134)	(43)	(123)	(119)	
Race: black (%)	22.8	31.1_a_	30.9_a_	19.6_ab_	11.0_b_	χ^2^(3)= 22.60, *p* < .001
	(139)	(70)	(21)	(33)	(15)	
Race: other^2^ (%)	11.3	13.8	13.2	11.9	5.9	χ^2^(3)= 5.63, *p* = .131
	(68)	(31)	(9)	(20)	(8)	
Spanish/Hispanic/Latino ethnicity (%)	13.7	15.6	16.2	11.3	12.5	χ^2^(3)= 1.93, *p* = .588
	(82)	(35)	(11)	(19)	(17)	
Married/living as married (%)	57.1	56.0	61.8	53.0	61.8	χ^2^(3)= 3.09, *p* = .378
	(341)	(126)	(42)	(89)	(84)	
Working full-or part-time (%)	41.2	37.8	36.8	43.5	46.3	χ^2^(3)= 3.47, *p* = .325
	(246)	(85)	(25)	(73)	(63)	
VA service-connected disability (%)	39.0	34.7	38.2	46.4	37.5	χ^2^(3)= 5.56, *p* = .135
	(233)	(78)	(36)	(78)	(51)	
Current psychiatric disorder (%)	74.9	79.1_a_	82.4_a_	73.2_a_	66.2_a_	χ^2^(3)= 9.18, *p* = .027
	(447)	(178)	(56)	(123)	(90)	
Past only psychiatric disorder (%)	37.7	37.3_ab_	30.9_ab_	31.0_a_	50.0_b_	χ^2^(3)= 13.63, *p* = .003
	(225)	(84)	(21)	(52)	(68)	
CAPS−5 PTSD severity (*M*)	39.7	39.4_b_	48.0_a_	40.2_b_	35.5_c_	*F*(3, 593)= 38.45, *p* < .001
	(8.6)	(8.6)	(6.7)	(8.2)	(6.7)	
CAPS−5 social impairment (*M*)	2.78	2.80_b_	3.06_a_	2.82_ab_	2.56_c_	*F*(3, 593)= 9.07, *p* < .001
	(0.69)	(0.69)	(0.62)	(0.65)	(0.71)	
CAPS−5 occupational impairment (*M*)	2.23	2.26_a_	2.53_a_	2.29_a_	1.96_b_	*F*(3, 593)= 5.08, *p* = .002
	(1.06)	(1.01)	(1.00)	(1.10)	(1.05)	
WHODAS (*M*)	2.62	2.67_a_	2.86_a_	2.70_a_	2.31_b_	*F*(3, 593)= 10.55, *p* < .001
	(0.78)	(0.81)	(0.80)	(0.72)	(0.72)	
WHOQOL-Bref psychological (*M*)	41.9	41.0_a_	38.3_a_	40.3_a_	47.3_b_	*F*(3, 593)= 5.85, *p* < .001
	(17.3)	(17.5)	(17.4)	(17.1)	(16.0)	
WHOQOL-Bref physical (*M*)	45.7	44.6_a_	40.1_a_	44.2_a_	52.0_b_	*F*(3, 593)= 8.64, *p* < .001
	(18.2)	(19.4)	(14.7)	(16.7)	(18.0)	
WHOQOL-Bref social (*M*)	41.9	39.9_a_	37.8_a_	41.2_a_	48.0_b_	*F*(3, 593)= 5.71, *p* < .001
	(20.7)	(20.4)	(21.1)	(20.4)	(20.3)	
WHOQOL-Bref environmental (*M*)	59.2	57.3_a_	56.4_a_	58.8_a_	64.3_b_	*F*(3, 593)= 6.49, *p* < .001
	(16.0)	(16.7)	(17.1)	(14.5)	(14.8)	

*Note*. PTSD = posttraumatic stress disorder. CAPS = Clinician-Administered PTSD Scale. WHODAS = World Health Organization Disability Adjustment Scale II (adapted; see measures). WHOQOL-Bref = World Health Organization Quality of Life-Bref. Race and ethnicity were self-reported, and participants could report multiple categories. For variables with a significant omnibus test, row means and percentages not sharing a common subscript differ at *p* < .05 or less after applying the Tukey-Kramer adjustment.

1 ANOVA F-test for continuous variables, Pearson’s χ2 for categorical variables.

2 Other race included Spanish/Hispanic/Latino, mixed races, and terms such as “human.”

**Fig. 1:**
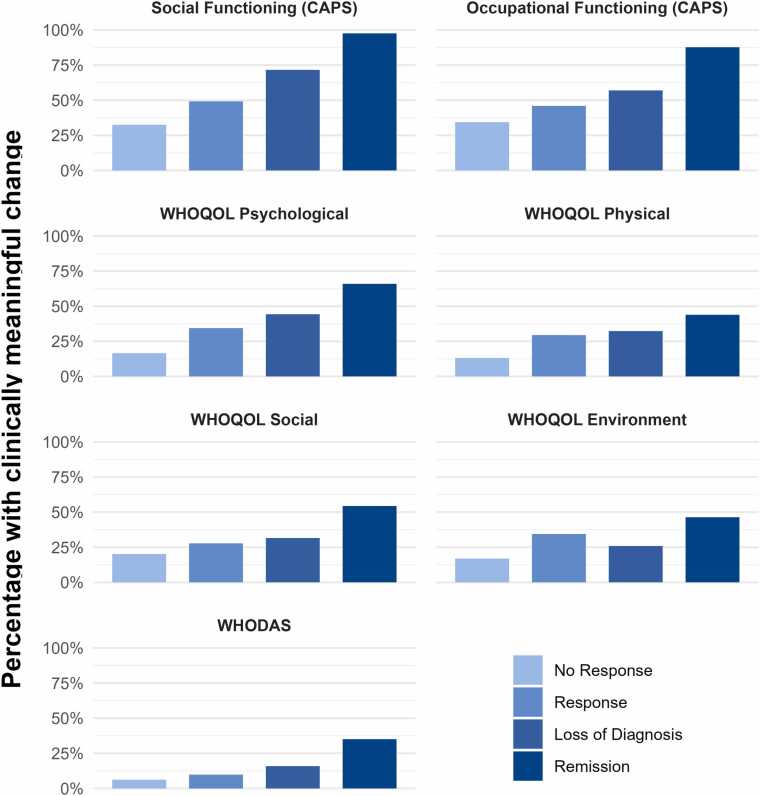
Percentage of participants in each symptom response group who had clinically meaningful improvement on each quality of life outcome.

Fig. 1 presents the percentage of participants in each group who experienced clinically meaningful change on each QoL outcome. The left side of Table 3 displays the results of successive between-groups comparisons for clinically meaningful change. Response, versus nonresponse, was associated with greater likelihood of improvement on all measures except the social domain of the WHOQOL-Bref and the WHODAS-II. Loss of diagnosis conferred no added benefit versus response, except on the CAPS Social Functioning scale. Remission, versus loss of diagnosis, was associated with greater likelihood of improvement on all measures.

**Table 3:** Adjusted Odds of Achieving Good Quality of Life Outcomes in PTSD Symptom Groups.

	Clinically Meaningful Improvement			Good Endpoint		
	Response vs. No Response	Loss of Diagnosis vs. Response	Remission vs. Loss of Diagnosis	Response vs. No Response	Loss of Diagnosis vs. Response	Remission vs. Loss of Diagnosis
WHOQOL Physical Health	2.61* (1.25, 5.39]	1.33 (0.67, 2.70)	2.31** (1.36, 3.96)	1.96 (0.55, 6.22)	2.04 (0.71, 6.86)	3.37*** (1.75, 6.62)
WHOQOL Psychological	3.19** (1.55, 6.57)	1.47 (0.74, 2.96)	3.57*** (2.07, 6.2526)	1.84 (0.45, 6.45)	1.95 (0.61, 7.68)	7.60*** (3.95, 15.20)
WHOQOL Social	1.61 (0.77, 3.32)	1.28 (0.62, 2.68)	3.76*** (2.18, 6.62)	1.32 (0.33, 4.42)	2.10 (0.68, 7.99)	4.556*** [2.40, 8.93)
WHOQOL Environment	2.93** (1.41, 6.11)	0.73 (0.36, 1.52)	3.97*** (2.26, 7.07)	3.59** (1.61, 8.09)	0.67 (0.31, 1.45)	4.57*** (2.53, 8.41)
WHODAS	1.71 (0.55, 4.96)	1.99 (0.75, 6.01)	5.95*** (3.08, 11.95)	1.18 (0.47, 2.77)	2.35* (1.02, 5.08)	3.80*** (2.15, 6.80)
CAPS Social Functioning	2.49** (1.33, 4.70)	2.71** (1.41, 5.26)	23.65*** (8.60, 85.27)	2.01 (0.67, 5.43)	4.30** (1.77, 12.16)	13.50*** (6.69, 29.89)
CAPS Occupational Functioning	2.11* (1.14, 3.93)	1.50 (0.80, 2.82)	5.75*** (3.13, 11.00)	1.23 (0.56, 2.57)	1.94 (0.94, 4.24)	8.96*** (4.82, 17.57)

*Note*. PTSD = posttraumatic stress disorder. CAPS = Clinician-Administered PTSD Scale. WHOQOL = World Health Organization Quality of Life-BREF. WHODAS = World Health Organization Disability Adjustment Scale-II (adapted; see measures). Table entries are odds ratios and 95% confidence intervals (below) from logistic regressions predicting each quality of life outcome from each successive PTSD symptom category, adjusting for baseline PTSD symptoms and the baseline score for that outcome. *p* < .05. **p < .01. ***p < .001.

**Fig. 2:**
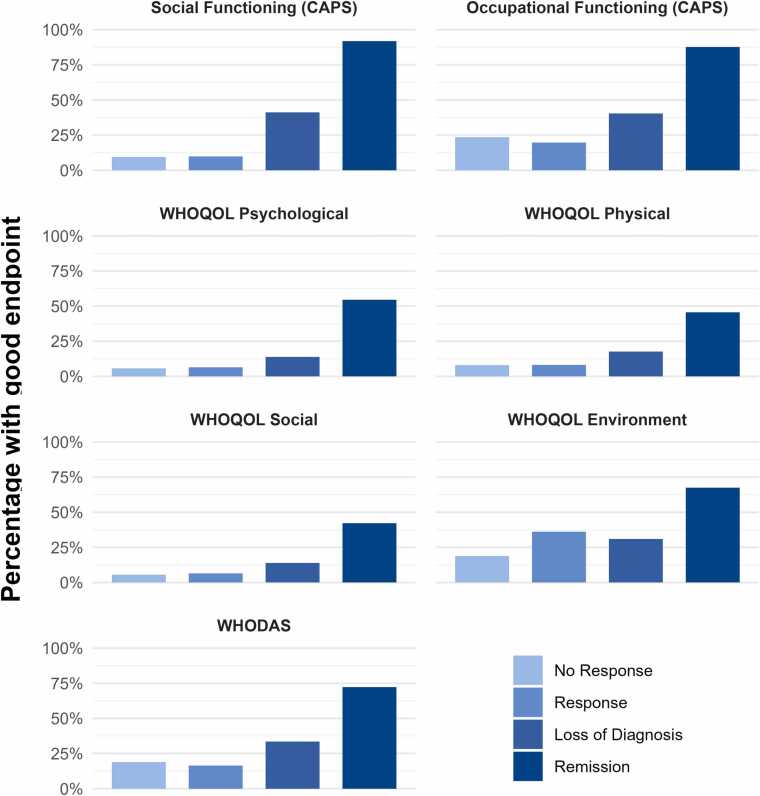
Percentage of participants in each symptom response group who achieved a good endpoint on each quality of life outcome.

Fig. 2 presents the percentage of participants in each group who attained a good endpoint on each QoL outcome. The right side of Table 3 displays the results of successive between-groups comparisons for achieving a good endpoint. In contrast to the findings for meaningful change, response was not associated with attaining a good endpoint for any measure except the WHOQOL-BREF environment domain. Loss of diagnosis, versus response, was associated with a good endpoint on CAPS−5 social functioning and the WHODAS. As in the analysis of meaningful change, remission, versus loss of diagnosis, was associated with a good endpoint on all measures.

## 4 Discussion

We used a novel approach to defining clinical significance by examining how symptom benchmarks of change in PTSD symptoms were associated with meaningful improvement and attaining a good endpoint in QoL. There were two key findings. First, although response only (without more substantial change) was associated with clinically meaningful change in QoL, it was not sufficient for helping participants attain good QoL endpoints. And second, remission increased the likelihood of meaningful change beyond loss of diagnosis and was necessary for attaining good endpoints.

Our findings for response, defined as a 10-point decrease in symptoms without greater symptom improvement, are consistent with the findings of the two prior studies that compared mutually exclusive groups [9], [10]. All three studies suggest that response alone is insufficient for promoting recovery. Our study differs from these other two in findings on remission versus loss of diagnosis, which was defined similarly in all studies. Whereas the two prior studies found that loss of diagnosis seemed like an optimal treatment goal, our study suggests that remission should be the goal instead. We found little evidence that loss of diagnosis was better than response alone. For example, on the two most rigorous outcomes (blinded clinician-rated social and occupational functioning on the CAPS−5), loss of diagnosis did not differ from response for clinically meaningful change on either measure or for attaining a good endpoint in occupational functioning. Loss of diagnosis was better than response in helping participants attain a good endpoint in social functioning, but remission conferred further advantage. In trying to understand possible reasons for the difference in findings other than sample characteristics, measures, and treatments studied, we note that the current study had more participants who experienced loss of diagnosis (51% vs. 29% [7] and 19% [8]) or remission (23% vs. 12% [7] and 6% [8]). Given our much larger sample, we had greater power than these other studies to find differences between response and loss of diagnosis. Replication in other samples may help clarify the optimal goal for treatment.

Descriptive analyses identified few demographic factors associated with PTSD response category. Older age and black race were associated with lesser response, and white race with better response. All QoL measures showed more consistent and robust findings indicating that individuals who remitted were more likely than all others to have better functioning and overall quality of life before treatment. This finding suggests that addressing these factors before treatment might improve outcomes, although it is also possible that other unmeasured factors are responsible, such as social determinants of health that could affect QoL. Another possible strategy would be to include treatment elements that directly target QoL as a way to improve overall outcomes.

When interpreting our findings, it is important to note that all participants were military veterans, who tend to be less responsive to PTSD treatment than non-Veterans [22]. Another limitation is that we studied only two treatments. Findings may differ for other treatments, both trauma-focused and non-trauma-focused psychotherapies and medications. Replication in non-veteran samples and with additional treatments is needed to further characterize the clinical benchmarks needed for recovery. Additional normative data on instruments such as the WHODAS [15] and WHOQOL-Bref [16] in US and other country-specific population samples would be beneficial as well. Results might differ if alternative definitions of symptom improvement were used, such as clinically significant change and minimal clinically important difference [4], [5], [23]. Other definitions, along with measures tailored for veteran samples [e.g., 24] may yield additional insights. However, besides the study’s large sample size, another strength is the pragmatic design, with clinically realistic patients and many therapists across 17 sites, which enhances generalizability to clinical practice. Furthermore, the use of mutually exclusive symptom change categories permitted identification of meaningful thresholds, e.g., between loss of diagnosis and remission.

The idea of defining symptom benchmarks of recovery aligns with a question posed by Kazdin years ago when he asked, “…will there be a way to provide a relatively simple, user-friendly, and hence feasible method(s) of assessing clinical significance for use in clinical research and practice?” [6, p. 458]. Building on prior studies [7], [8], [9], [10], we tested his suggestion to validate categories of symptom change using measures of quality of life. Our findings add to evidence that we need to focus on more than symptom change when setting treatment goals for patients and for evaluating treatments because it is clear that symptom change is not enough. We propose that recovery should be the goal: a process of change through which individuals improve their health and wellness, live self-directed lives, and strive to reach their full potential [25].

The question is how to help patients experience full recovery. There are many possible options: adding treatments that target QoL [26]; using a precision medicine approach to identify the optimal treatment for a given patient [27]; tailoring treatment to address a patient’s specific needs [28]; promoting patient engagement through shared decision making [29]; and novel approaches such as psychedelic-assisted therapy [30]. None of these approaches has yielded strong evidence that they alone promote recovery. But all have promise. We suggest that using consistently defined symptom benchmarks in treatment outcome research can yield more definitive answers and make a difference in patients’ lives.

## Declaration of Competing Interest

The authors declare the following financial interests/personal relationships which may be considered as potential competing interests: Paula Schnurr reports a relationship with Healing Breakthrough that includes: funding grants. Paula Schnurr is listed as an Editorial Board member of JMAD. If there are other authors, they declare that they have no known competing financial interests or personal relationships that could have appeared to influence the work reported in this paper.
